# Inhibition of Antigen-Specific and Nonspecific Stimulation of Bovine T and B Cells by Lymphostatin from Attaching and Effacing Escherichia coli

**DOI:** 10.1128/IAI.00845-16

**Published:** 2017-01-26

**Authors:** Robin L. Cassady-Cain, Elizabeth A. Blackburn, Charlotte R. Bell, Elizaveta Elshina, Jayne C. Hope, Mark P. Stevens

**Affiliations:** a The Roslin Institute and Royal (Dick) School of Veterinary Studies, University of Edinburgh, Midlothian, United Kingdom; b The Centre for Translational and Chemical Biology, University of Edinburgh, Edinburgh, United Kingdom; University of Michigan

**Keywords:** lymphostatin, lymphocyte, inhibition, adaptive, immunity, Escherichia coli, cell proliferation, host-pathogen interactions, adaptive immunity, inhibition, lymphocytes

## Abstract

Enterohemorrhagic Escherichia coli (EHEC) and enteropathogenic E. coli (EPEC) are enteric bacterial pathogens of worldwide importance. Most EPEC and non-O157 EHEC strains express lymphostatin (also known as LifA), a chromosomally encoded 365-kDa protein. We previously demonstrated that lymphostatin is a putative glycosyltransferase that is important in intestinal colonization of cattle by EHEC serogroup O5, O111, and O26 strains. However, the nature and consequences of the interaction between lymphostatin and immune cells from the bovine host are ill defined. Using purified recombinant protein, we demonstrated that lymphostatin inhibits mitogen-activated proliferation of bovine T cells and, to a lesser extent, proliferation of cytokine-stimulated B cells, but not NK cells. It broadly affected the T cell compartment, inhibiting all cell subsets (CD4, CD8, WC-1, and γδ T cell receptor [γδ-TCR]) and cytokines examined (interleukin 2 [IL-2], IL-4, IL-10, IL-17A, and gamma interferon [IFN-γ]) and rendered T cells refractory to mitogen for a least 18 h after transient exposure. Lymphostatin was also able to inhibit proliferation of T cells stimulated by IL-2 and by antigen presentation using a Theileria-transformed cell line and autologous T cells from Theileria-infected cattle. We conclude that lymphostatin is likely to act early in T cell activation, as stimulation of T cells with concanavalin A, but not phorbol 12-myristate 13-acetate combined with ionomycin, was inhibited. Finally, a homologue of lymphostatin from E. coli O157:H7 (ToxB; L7095) was also found to possess comparable inhibitory activity against T cells, indicating a potentially conserved strategy for interference in adaptive responses by attaching and effacing E. coli.

## INTRODUCTION

Enterohemorrhagic Escherichia coli (EHEC) is associated with hemorrhagic colitis and hemolytic-uremic syndrome in humans, and cattle are a key reservoir of infection. Enteropathogenic E. coli (EPEC) shares many features with EHEC and is a major cause of acute diarrhea in infants in developing countries. Both pathotypes colonize intestinal mucosa via the formation of attaching and effacing (AE) lesions in a manner that requires a type III protein secretion system (T3SS), as well as accessory virulence factors (1). One such factor is lymphostatin (also known as LifA), a chromosomally encoded protein with a predicted molecular mass of 365 kDa that is expressed by most EPEC and non-O157 EHEC strains (2). Lymphostatin was first described for EPEC O127:H6 as a factor required for inhibition of mitogen-activated proliferation of human peripheral blood monocytes (PBMCs) (2), an activity that had also been observed with murine splenic and mucosal lymphocytes treated with EPEC lysates (3). Lymphostatin was recently reported to be a secreted effector of the T3SS (4); however, lymphostatin activity does not require injection of the protein into cells, as it can be demonstrated with a T3SS-negative E. coli K-12 strain bearing *lifA* on a cosmid (2) and detected using purified protein (5). Separately, a factor nearly identical to LifA was reported to mediate adherence of EHEC O111:H^−^ to cultured epithelial cells (EHEC factor for adherence [Efa1]) (6), and mutations in the gene impaired type III secretion in some strains (7, 8). We previously demonstrated that lymphostatin is required for intestinal colonization of calves by non-O157 EHEC serogroups O5, O111 (7), and O26 (8); however, the extent to which this reflects a role in modulation of bovine immune responses, adherence, or indirect effects on type III secretion remains ill defined. Lymphostatin has also been shown to promote colonization of the murine intestines and colonic hyperplasia by the attaching and effacing pathogen Citrobacter rodentium (9).

Lymphostatin exhibits N-terminal homology with large clostridial toxins, including a conserved glycosyltransferase domain and predicted DXD catalytic motif (6). Progress in understanding the mode of action of the protein was previously hindered by the instability of plasmid clones and suspected protein toxicity; however, we recently developed an inducible system for affinity purification of LifA (5). Using site-directed mutagenesis, we observed that the DXD motif is required for lymphostatin activity and for binding of UDP-*N*-acetylglucosamine (UDP-GlcNAc), indicating that it may act by GlcNAc modification of cellular factors. The EHEC O157:H7 serotype that is predominantly associated with human disease in North America and Europe typically lacks lymphostatin; however, sequencing of the prototype strain revealed that a homologue is encoded on the pO157 virulence plasmid (*toxB* or *l7095* [10]) that has subsequently been found in many EHEC and EPEC strains (11–13) and proposed to be type III secreted (4). ToxB exhibits 29.2% identity (and 62.3% similarity [14]) at the amino acid level to LifA using the full amino acid sequence, and a closer examination of the first 1,033 amino acids (aa) (encompassing the glycosyltransferase domain) shows a higher identity, 36.4% (and 68.7% similarity). It was reported that E. coli O157:H7 has a lymphostatin-like activity that was absent upon curing of the ca. 92-kb pO157 plasmid (2). However, plasmid pO157 encodes other putative virulence factors, and a significant role for *toxB* in inhibition of lymphocyte proliferation could not be detected with a *toxB* deletion mutant, albeit using an insensitive assay reliant on crude bacterial lysates (15). Certain Chlamydia species also contain a family of lymphostatin homologues which have been implied to act as cytotoxins (16).

Lymphostatin activity does not appear to be host restricted, having been detected with mitogen-activated peripheral blood monocytes from humans (2), mice (9), and calves (7). However, relatively little is known about whether it acts on specific cell subsets and the sensitivity of the effect to stimulus (e.g., mitogens, antigens, or cytokines). This is particularly pertinent in relation to colonization of the bovine reservoir host, where modulation of innate and adaptive responses is likely to play a role in bacterial persistence. We therefore investigated the activity of recombinant LifA against bovine T, B, and NK cells and lymphocyte subsets stimulated with various agonists. We suggest that lymphostatin acts as a global T cell inhibitor, possibly by conditioning T cells to be functionally unresponsive, as treated cells remain refractory to mitogen for many hours after transient exposure. We also observed that lymphostatin blocks mitogen-activated secretion of cytokines and, for the first time, stimulation induced by antigen presentation to autologous lymphocytes. We successfully cloned, expressed, and affinity purified full-length ToxB from E. coli O157:H7 and definitively showed that it possesses lymphostatin-like activity. This suggests a potentially conserved strategy among AE E. coli to interfere with adaptive immune response and adds to the relatively small number of bacterial factors described to directly target adaptive immune function.

## RESULTS

### Full-length lymphostatin is a selective inhibitor of T and B lymphocyte activation.

**FIG 1:**
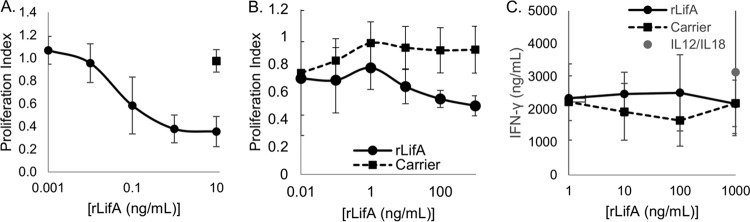
Effect of recombinant lymphostatin on proliferation of primary bovine T, B, and NK cells. (A) Primary T cells were stimulated with ConA for 72 h, and proliferation was measured using a colorimetric assay. Data are averages ± standard deviations from 8 donors. The ED_50_ was 54 pg/ml (±19 pg/ml) (138 fM). The purity of the population was >85% by flow cytometry. The ConA response was unaffected by treatment with protein carrier buffer (solid square). (B) Primary B cells were stimulated with recombinant IL-4, and lymphostatin where indicated, for 72 h and proliferation measured using a colorimetric assay. Data are averages ± standard deviations from 4 independent donors. The ED_50_ was 11 ng/ml (±14 ng/ml) (30 pM).The purity of the population was >97% by flow cytometry. (C) Primary NK cells were stimulated with recombinant IL-12 and IL-18 overnight. The purity of the cell population was >85%. Production of IFN-γ was measured in a sandwich ELISA against a recombinant protein standard. The amount of IFN-γ produced by treatment with IL-12 and IL-18 alone is shown as a single data point to indicate the maximum IFN-γ release possible. The limit of detection was 2 ng/ml. Data are averages ± standard deviations from 3 independent donors. Data are plotted with a log scale on the *x* axis for all charts.

Recently, we showed that recombinant full-length lymphostatin was able to potently inhibit mitogen-stimulated T cell proliferation, with a 50% effective dose (ED_50_) in the femtomolar range in the absence of direct cytotoxic effects (5). Given that all previous examination of the effect of lymphostatin has been in bulk PMBC preparations using predominantly T cell-affecting mitogens, we wished to determine whether the effect of lymphostatin is restricted to T cells or whether other lymphocytes might also be affected. To that end, we compared the effects of lymphostatin on stimulation of T cells, B cells, and NK cells. Data obtained for T cells were essentially as reported previously, with a clear sigmoidal dose-response curve and an ED_50_ of 54 pg/ml (±19 pg/ml) (Fig. 1A). Incubation of B cells with lymphostatin, followed by stimulation by interleukin 4 (IL-4), an activator of B cell proliferation, showed that lymphostatin induced a reduction in the proliferative capacity of B cells compared to that of the control (Fig. 1B; black circles indicate treatment with lymphostatin, and black squares indicate treatment with a similar concentration of lymphostatin protein buffer). The effect was concentration dependent and was titrated out by 1 ng/ml (Fig. 1B). The ED_50_ of recombinant LifA (rLifA) on B cells was calculated to be 11 ng/ml (±14 ng/ml; 30 pM), about 200-fold lower than the ED_50_ for T cells. In contrast, lymphostatin had little or no effect on the production of gamma interferon (IFN-γ) by NK cells stimulated with IL-12 and IL-18 (Fig. 1C). IL-12 and IL-18 are able to potently induce NK cells to produce high levels of IFN-γ. Although the IFN-γ production was lower in the presence of rLifA or carrier than that of IL-12 or IL-18 alone (Fig. 1C, single gray circle), no significant differences were detected.

### Effect of lymphostatin on bovine T cell subsets and cytokine production.

**FIG 2:**
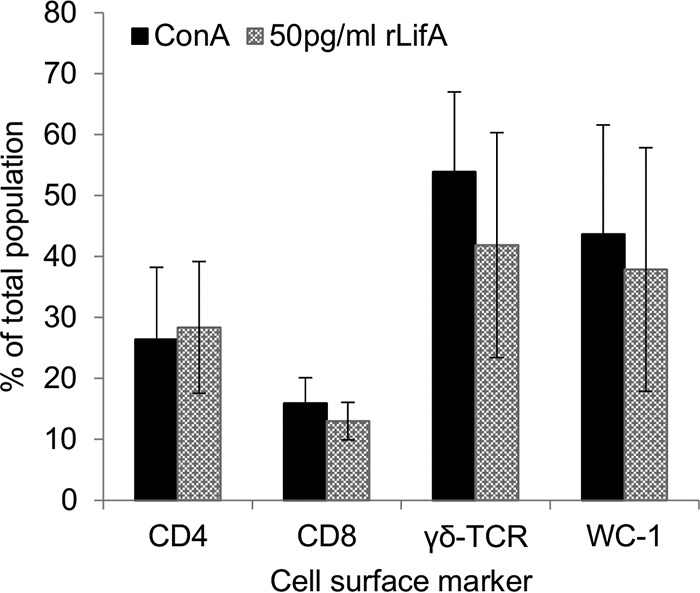
Effect of subsaturating concentrations of lymphostatin on the abundance of T cell subsets after ConA-stimulated proliferation. Cells were treated with either ConA with protein carrier buffer or ConA plus 50 pg/ml of rLifA at a concentration similar to the ED_50_ for lymphostatin in ConA-stimulated proliferation. Cells were harvested at 72 h, stained for the indicated surface markers, and measured by flow cytometry. The averages ± standard deviations from four independent donors are shown. There were no statistically significant differences for any of the markers tested between the groups treated with ConA and ConA plus rLifA for any of the markers tested (paired *t* test, *P* > 0.05).

**FIG 3:**
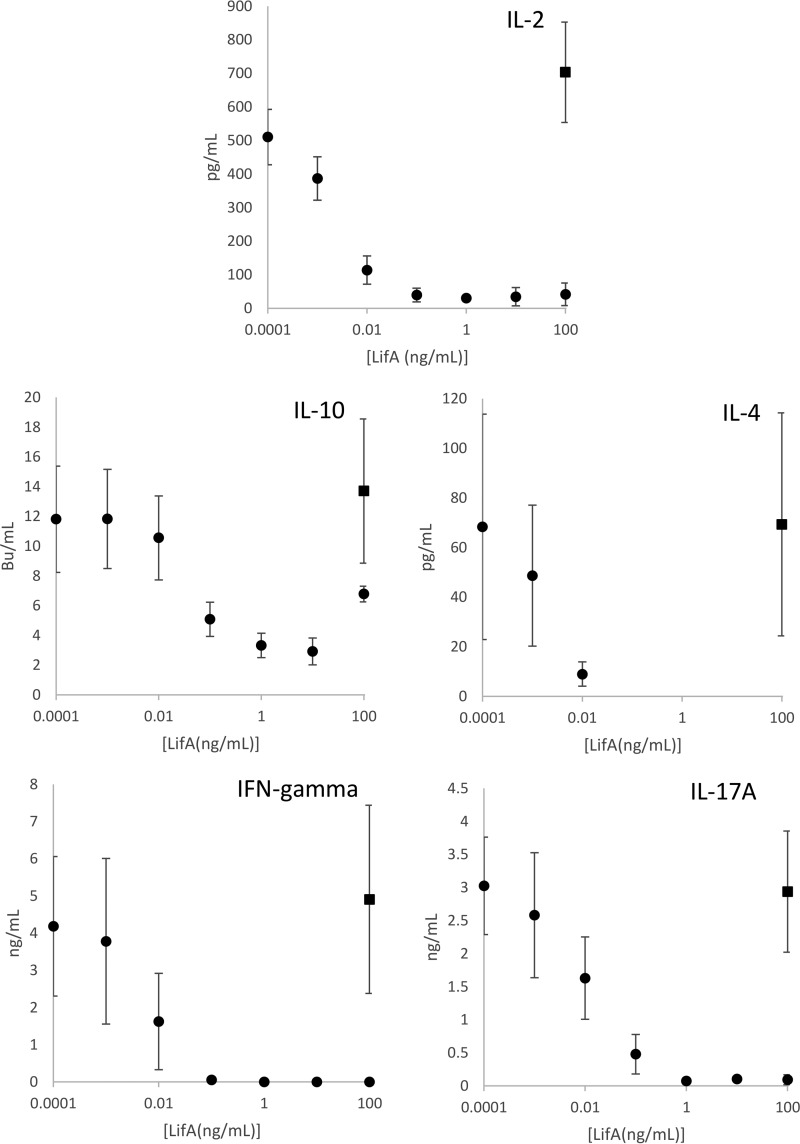
Cytokine secretion from bovine T cells treated with lymphostatin and stimulated with ConA. Cytokine secretion was measured at 24 h poststimulation in cell culture supernatants using quantitative sandwich ELISAs. The averages ± standard deviations from 3 independent donors are shown. Data from samples treated with lymphostatin are indicated by solid circles. The maximum secretion expected is from cells treated with ConA and protein carrier buffer (but no lymphostatin). The values obtained for these samples are indicated by a solid square over the 100 ng/ml marker and are stated in Results. The ED_50_ required to inhibit cytokine production was not significantly different from the ED_50_ required to inhibit lymphocyte proliferation in all instances examined (one-way analysis of variance [ANOVA] and *post hoc* Tukey test, *P* > 0.05). The limits of detection for each ELISA were as follows: IL-2, 40 pg/ml; IL-4, 4 pg/ml; IL-10, 2 BU/ml; IFN-γ, 2 ng/ml; and IL-17A, 188 pg/ml.

Having established that the effect of lymphostatin is most potent on T lymphocytes, we sought to understand whether it has a global effect across the T cell compartment, or if it preferentially affects a specific subset of T cells. First, the percentage of cells expressing CD4, CD8, WC-1 (a coreceptor of γδ T cell receptor [γδ-TCR]), and γδ-TCR was assessed by flow cytometry, using an enriched T cell preparation treated with a subsaturating concentration of rLifA (approximately the ED_50_) and concurrent stimulation with concanavalin A (ConA). These cells reflect the main T lymphocyte subsets present in bovine peripheral blood. In comparison to the controls treated with ConA alone, there was no statistically significant change in the proportions of any of the T cell populations tested (paired *t* test; *P* values > 0.05 [Fig. 2]). To further probe this effect, we quantified the secretion of a number of T cell-derived cytokines in response to ConA stimulation of an enriched T cell population that can broadly be used to reflect Th1, Th2, regulatory T cell (Treg), and Th17 subset populations. ConA-stimulated lymphocytes (not treated with lymphostatin) secreted IL-2 (703 ± 149 pg/ml), IL-4 (69 ± 45 pg/ml), IL-10 (13.7 ± 4.8 biological units [BU]/ml), IFN-γ (5 ± 3 ng/ml), and IL-17A (3 ± 1 ng/ml) above the level of detection in all cases (Fig. 3, solid black squares). Secretion of all of the cytokines measured showed a dose-dependent decrease in response to increasing concentrations of lymphostatin, mirroring the proliferation data previously measured (Fig. 3). In most cases, cytokine secretion was below or close to the limit of detection by the methods used following treatment with rLifA at 1 ng/ml or higher.

### Pretreatment of T cells with lymphostatin induces long-lived resistance to mitogenic activation.

**FIG 4:**
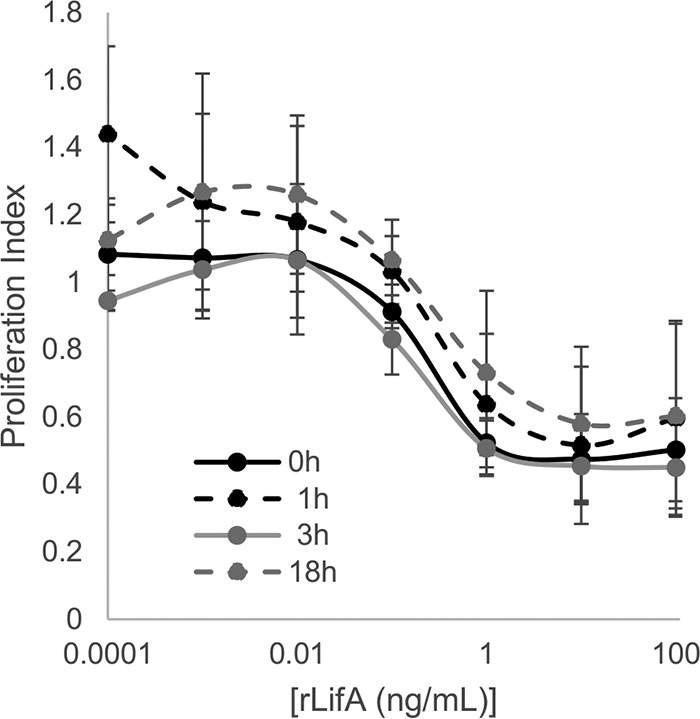
Transient pretreatment of primary bovine T cells with lymphostatin induces lasting inhibition of the response to ConA. Primary T cells were pretreated with rLifA, protein was washed away, and cells were stimulated with 1 μg/ml of ConA at the times indicated. Proliferation was measured by colorimetric assay. Data represent averages ± standard deviations from 4 independent donors. Data are plotted with a log scale on the *x* axis.

In the assays reported so far, lymphostatin was present in the medium for the duration of culture. To determine if transient exposure of lymphocytes to lymphostatin was sufficient to render them refractory to mitogenic stimulation, we pretreated T cells with a range of protein concentrations (100 ng/ml to 0.1 pg/ml for 1 h) and then washed the cells to remove the protein and added ConA immediately or 1, 3, or 18 h after withdrawal of rLifA. In all cases, concentration-dependent inhibition of lymphocyte proliferation was observed after transient pretreatment of cells (Fig. 4). Even 18 h after withdrawal of lymphostatin at doses of 1 ng/ml or higher, the T cells were inhibited from proliferating in the presence of ConA.

### Lymphostatin inhibits lymphocyte stimulation via antigen presentation.

**FIG 5:**
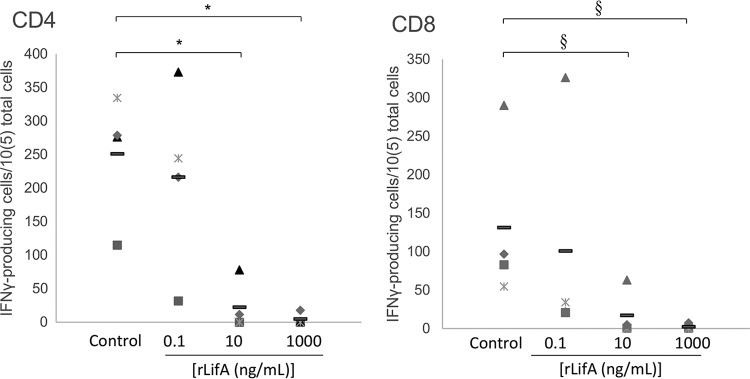
Effect of lymphostatin on stimulation via antigen presentation to defined bovine T cells. Data from 4 independent experiments, each with technical replication in triplicate, are shown for both CD4^+^ and CD8^+^ T cells. Each symbol refers to the mean of an individual experiment, with the overall mean indicated by the solid black bar. “Control” refers to antigen stimulation of the T cells where protein carrier buffer was included but not protein (this is the maximum number of cells that would be expected to be measured in the assay). A minimum spot size of 15 and intensity of 5 were used for analysis. *, *P* < 0.05; §, *P* = 0.1 (compared to carrier control; paired *t* test).

In order to establish whether the effect of lymphostatin on lymphocyte proliferation is specific to inhibition of mitogenic stimulation, we evaluated the ability of lymphostatin to inhibit antigen-stimulated activation of bovine CD4 and CD8 T cells. We exploited an established assay in which antigen-presenting cells (APCs) sustained in culture and permanently infected with the protozoan parasite Theileria parva were used to present antigens *in vitro* to autologous T. parva-specific T cell populations from cattle that had been rendered immune to T. parva by simultaneous infection and treatment (a method for vaccination against the parasite [17, 18]). Activation of the T cells in response to recognition of antigens presented by the T. parva-infected cells in culture was quantified by their ability to secrete IFN-γ using an enzyme-linked immunosorbent spot (ELISPOT) assay. As in earlier assays, T. parva-specific T cells and infected cells presenting T. parva antigens were incubated with a range of concentrations of lymphostatin or the carrier control (Fig. 5). For both CD4 cells and CD8 cells, a lymphostatin concentration-dependent inhibition of the number of IFN-γ-producing cells was measured by ELISPOT assay compared to that in an untreated control (which indicates the maximum number of affected cells expected in the assay). The effect with CD4^+^ cells was very clear and showed a similar titration to inhibition of ConA-mediated stimulation, with both the 10-ng/ml and 1-μg/ml treatments being statistically significantly different from the carrier control (*P* < 0.05). The effect with CD8^+^ cells was similar; however, the number of IFN-γ-producing cells was lower, and the variation between experiments was higher, meaning that the difference did not reach statistical significance (*P* = 0.1). Nonetheless, the trend within each individual replicate mirrors the results obtained with the CD4^+^ cells, with lymphostatin inhibiting antigen-induced IFN-γ secretion by bovine CD4^+^ and, likely, CD8^+^ T cells, which is consistent with the data obtained for T cell subsets and cytokine production in mitogen-stimulated T cell populations.

### IL-2-induced expansion of T cells can be inhibited by lymphostatin.

**FIG 6:**
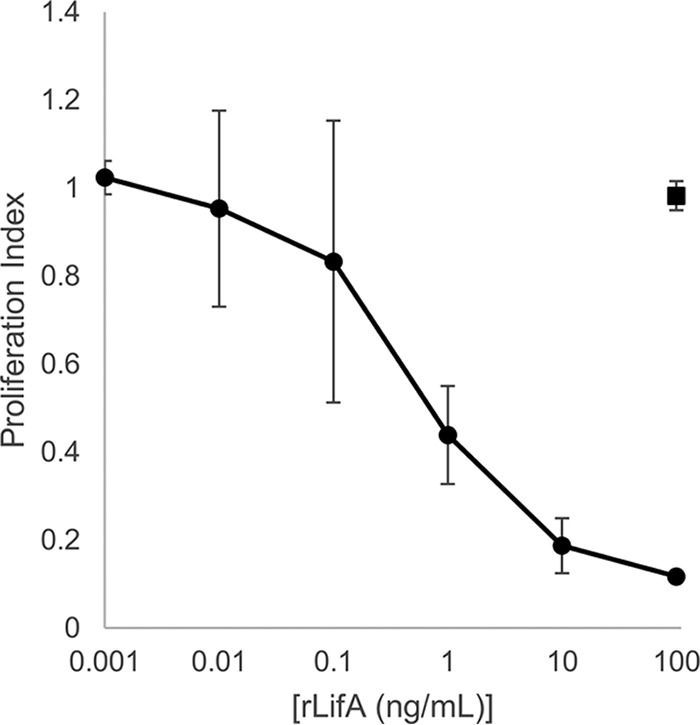
Effect of lymphostatin on IL-2-stimulated proliferation of primary bovine T cells. PBMCs were initially stimulated with ConA. After 4 days, cells were treated with rLifA at the final concentrations indicated, followed immediately by stimulation with IL-2 for a total of 72 h. Proliferation was measured using a colorimetric assay. ED_50_ = 500 pg/ml (± 290 ng/ml) (1.4 pM). Data from 4 independent donors are shown. Cells with no stimulus, cells stimulated with IL-2 but no protein, and cells treated with protein carrier buffer and stimulated with IL-2 were included as controls. The IL-2 response is unaffected by protein carrier buffer (solid square). Data are plotted with a log scale on the *x* axis.

Given that IL-2 is the major growth factor for induction of activation and expansion of the T cell compartment, we examined whether lymphostatin was able to interfere directly in IL-2 signaling during the assays, or whether its effects are restricted to the initiation of proliferation. T cells were treated with a range of lymphostatin concentrations and driven to proliferate with IL-2. Clear concentration-dependent inhibition of IL-2-stimulated proliferation of bovine T cells was observed (Fig. 6), with an ED_50_ calculated to be 500 pg/ml (±290 ng/ml) (1.4 pM).

### PMA and ionomycin stimulation of T cells is not affected by lymphostatin.

**FIG 7:**
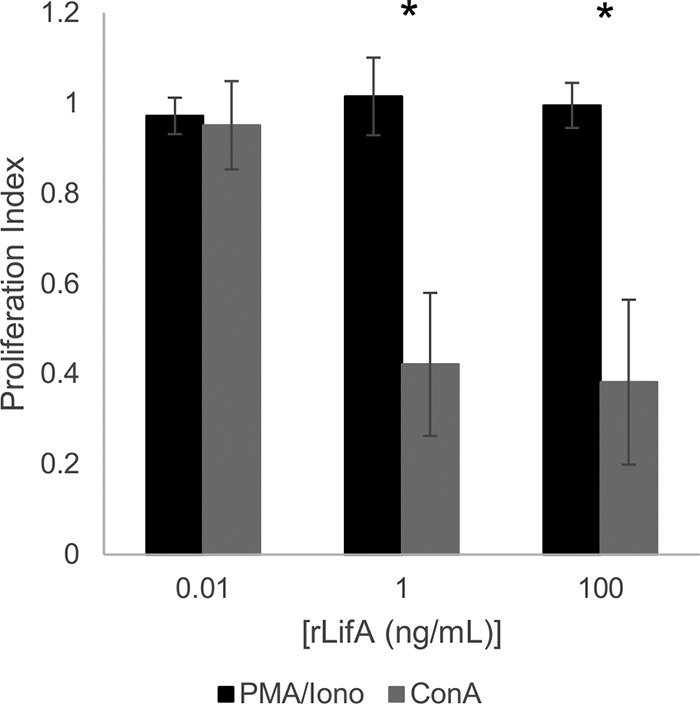
Effect of lymphostatin on PMA- and ionomycin (Iono)-stimulated proliferation of primary bovine T cells. Primary T cells were treated with rLifA at the final concentrations indicated and stimulated with 50 ng/ml of PMA and 1 μg/ml of ionomycin for 72 h. Proliferation was measured using a colorimetric assay. Averages ± standard deviations from 3 independent donors are shown. Data are plotted with a log scale on the *x* axis. The difference in ConA and PMA and ionomycin responses was significant at two rLifA concentrations (*, *P* ≤ 0.05 at 1 and 100 ng/ml comparing PMA and Iono to ConA). Cells without treatment, cells treated with ConA or PMA and ionomycin, and cells treated with protein carrier buffer and ConA or PMA and ionomycin were included as controls.

In order to investigate the stage at which lymphostatin affects T cell signaling and disrupts the proliferative program, we queried whether rLifA was able to inhibit proliferation stimulated using phorbol 12-myristate 13-acetate (PMA) and ionomycin. PMA activates protein kinase C, while ionomycin is a calcium ionophore. Together they mimic TCR and coreceptor activation, but in a way that bypasses membrane receptor signaling (19). Lymphostatin was not able to inhibit PMA- and ionomycin-induced T cell proliferation (Fig. 7), in contrast to controls stimulated with ConA, which were significantly impaired in proliferation at 1 ng/ml and 100 ng/ml of rLifA (*P* ≤ 0.05), indicating that lymphostatin may interfere with membrane-proximal signaling or pathways dependent on such.

### The lymphostatin homologue ToxB from E. coli O157:H7 also inhibits ConA-stimulated T cell proliferation.

**FIG 8:**
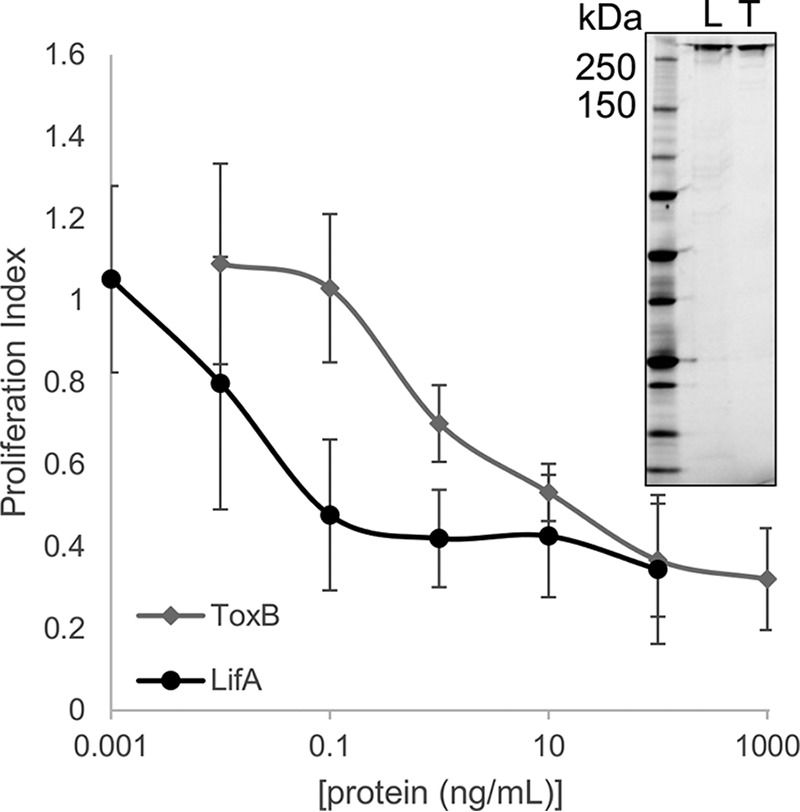
Effect of recombinant ToxB on mitogenic stimulation of primary bovine T cells. Primary T cells were treated with either recombinant ToxB or LifA, followed by immediate addition of ConA for a total of 72 h, and proliferation was measured using a colorimetric assay. The ED_50_ was calculated to be 1.1 ng/ml (±0.88 ng/ml) (2.8 pM) for ToxB, compared to 0.013 ng/ml (±0.01 ng/ml) (0.03 pM) for LifA. Averages ± standard deviations from 4 independent donors are shown. Data are plotted with a log scale on the *x* axis. (Inset) L, LifA; T, ToxB. In a comparison of the effect of ToxB to that of LifA, the ED_50_ is not statistically significantly different. Cells without treatment, cells treated with ConA, and cells treated with protein carrier buffer and ConA were included as controls.

While it is clear that lymphostatin is a potent inhibitor of bovine lymphocyte function and a key colonization factor of serogroup O5, O26, and O111 strains in calves (7, 8), it is absent from most serogroup O157 strains, which are prevalent in ruminants in many parts of the world and an important cause of zoonotic diarrheal illness in humans. A homologous protein (ToxB) is encoded by most EHEC O157 strains, but definitive evidence of a role in modulating lymphocyte function is lacking. We cloned the full-length ToxB protein in a tightly inducible prokaryotic expression system, affinity purified the protein to ca. 90% purity (Fig. 8, inset) and evaluated its ability to inhibit ConA-stimulated proliferation of bovine T cells relative to rLifA. Recombinant ToxB inhibited ConA-stimulated proliferation of bovine T cells in a concentration-dependent manner (Fig. 8), with an ED_50_ of 1,100 pg/ml (±880 pg/ml) (2.8 pM) for ToxB, compared to 10 pg/ml (±10 pg/ml) (0.03 pM) for rLifA. This represents about a 100-fold difference in ED_50_; however, ToxB was still able to inhibit proliferation of T cells in the picomolar range of concentrations.

## DISCUSSION

Lymphostatin plays an important role in intestinal colonization of calves by non-O157 EHEC (7) and in persistence of C. rodentium in murine intestines and induction of colonic hyperplasia (9). It has been hypothesized that this may reflect its ability to interfere with lymphocyte proliferation and proinflammatory cytokine synthesis. Previous studies have mostly utilized crude peripheral blood monocyte populations to demonstrate LifA activity (2, 7, 8, 20, 21), and the extent to which lymphostatin acts on specific cell populations and can inhibit stimulation via distinct agonists and pathways has received little attention. Moreover, published assays have relied on crude lysates of E. coli producing lymphostatin, in which activity can be hard to separate from the inhibitory effects of other constituents. Indeed, inhibitory effects of lysates of nonpathogenic E. coli strains lacking LifA have been detected on human or bovine PBMCs when used at higher concentrations (2, 8).

In this study, using cells from the bovine reservoir of EHEC, we demonstrated that highly purified lymphostatin predominantly acts on the T cell compartment, with near complete inhibition of proliferation of all major T cell subsets in the femtomolar range. As previously reported, no evidence that lymphostatin is directly cytotoxic to target cells exists (5). The observed inhibition of cytokine production by LifA-treated bovine T cells is consistent with previously published data (2) showing that expression of IL-2, IL-4, and IFN-γ was reduced by treatment of ConA-stimulated human PBMCs with lymphostatin. The latter study differed by measuring cytokine mRNA levels rather than secreted proteins, and IL-10 and IL-17 were not assessed. From these results, we would conclude that it seems likely that lymphostatin has a global effect on the T cell compartment, without bias for a particular cell subset or type of response (i.e., Th1 versus Th2), at least under the conditions tested in this study. It would be of interest to confirm this hypothesis *in vivo*; however, analysis of immune responses induced by *lifA* mutants is likely to be confounded by the attenuating effect of the mutation, such that cytokine responses are likely to be affected by both the presence of lymphostatin and the bacterial load acting on the immune system. As *lifA* is required for intestinal colonization of mice by C. rodentium, it would be of interest to determine if the role of lymphostatin in bacterial persistence is still observed when lymphocyte subsets are removed by mutagenesis or antibody-mediated depletion.

Lymphostatin also inhibited IL-4-mediated activation of bovine B cells, but to a lesser extent than with ConA-treated T cells, with higher LifA concentrations being required to detect equivalent inhibition. Bovine NK cells were insensitive to lymphostatin, at least in the context of IFN-γ induction under the assay conditions. The basis of cell-type-specific responses to LifA requires further study and may reflect differences in receptor availability and/or the pathways or molecules on which lymphostatin acts. It will also be of interest to explore if lymphostatin differentially affects activated and differentiated subsets of lymphocytes, although such populations can be difficult to consistently establish *in vitro*.

For the first time, we have demonstrated that lymphostatin is able to inhibit antigen-stimulated proliferation of bovine CD4^+^ T cells. This is significant in the context of natural infections, and future experiments could consider if adaptive immune responses of cattle to EHEC-expressed antigen(s) are sensitive to the presence or absence of lymphostatin. However, as noted above, this is complicated by the fact that lymphostatin influences intestinal colonization by EHEC, and thus, total exposure to antigens can be expected to be lower upon infection with *lifA* mutants relative to the wild-type strain. We previously examined the phenotype of intraepithelial lymphocytes (IEL) exposed to Shiga toxin-producing E. coli O103 in situ in a bovine ligated intestinal loop model in which equivalent densities of wild-type and mutant bacteria can be instilled into segments of the gut (22). No significant effects on proliferative capacity, NK cell activity, or cytokine transcript profile were detected on exposure to EHEC in these studies (22). The strain used was positive for the *lifA* gene by PCR, but it is unknown whether the full-length protein was expressed under the assay conditions, and truncated variants of *lifA* exist in some AE E. coli organisms. It is noteworthy that attenuation of *lifA* mutants of EHEC in calves or C. rodentium in mice can be detected before adaptive responses may be expected to have developed. Further, a DXD substitution that ablated lymophostatin activity against bovine PBMCs (8) and T cells (5) did not significantly attenuate an EHEC O26:H^−^ strain in a calf intestinal colonization model, indicating that the immunomodulatory role of LifA may not be strictly necessary for persistence of EHEC in cattle (8), though it should be stressed that adaptive immune responses and long-term persistence were not investigated in this study. It is possible that the attenuation of *lifA* mutants reflects the additional putative role of lymphostatin as an adhesin and/or indirect effects on the expression and secretion of type III secreted proteins as has been observed in some strains. Moreover, recent evidence indicates that LifA is itself an effector of the T3SS, and its role(s) once injected into cells is not fully understood. The availability of highly purified lymphostatin offers scope to revisit activity of the protein against constituents of the mucosal immune system and development of adaptive immune responses to pathotypes of E. coli in situ.

The inhibitory effect of lymphostatin on T cells was observed when cells were stimulated via the IL-2 receptor with soluble IL-2, or with the mitogens ConA or pokeweed mitogen (20), but not following stimulation with PMA and ionomycin. Given that PMA and ionomycin stimulation bypasses membrane receptor signaling, this implies that lymphostatin likely perturbs the activity of a molecule(s) important in early signaling events. Given the intricacy and complex interconnections between signaling pathways in T cell activation, it is difficult to speculate on the level at which lymphostatin exerts its activity. However, the fact that lymphostatin inhibits ConA stimulation, as well as IL-2-stimulated proliferation, but not PMA and ionomycin makes the membrane-proximal signaling events that feed into the mitogen-activated protein kinase (MAPK) pathway attractive targets for further investigation. The murine model of C. rodentium colonization (9) and published sensitivity of murine lymphocytes to LifA (9) offer opportunities to examine the role of lymphostatin when specific signaling pathways are ablated via gene knockouts or inhibitors.

Our recent finding that lymphostatin binds UDP-GlcNAc in a manner that requires a DXD motif that is also required for inhibitory activity (5) supports predictions from sequence homology and structural modeling that lymphostatin acts as a glycosyltransferase. It is noteworthy that our study found that T cells are rendered refractory to mitogen for at least 18 h after transient exposure to lymphostatin, indicating that LifA may rapidly act on T cells and that any modification(s) has a lasting effect. Studies on the binding of LifA to lymphocytes, whether uptake occurs via specific pathways, and whether LifA requires processing in order to act on its cellular target are warranted and may help to explain differences in cell sensitivity. Indeed, it is noteworthy that lymphostatin is predicted to contain a cysteine protease motif, which in the case of large clostridial toxins is required for autocatalytic cleavage of the toxin following its insertion in the endosome membrane in order to release the catalytic domain into the cytosol. Our current research aims to identify cellular proteins that interact with lymphostatin and whether they act as GlcNAc acceptors.

It is striking that despite the role of lymphostatin in intestinal colonization by various attaching and effacing pathogens, it is absent in the vast majority of serogroup O157 strains that are prevalent in Europe and America. A homologue encoded by the pO157 virulence plasmid was proposed to be functionally equivalent to LifA based on loss of lymphostatin-like activity against ConA-stimulated human PBMCs upon curing of pO157 (2). However, pO157 encodes other secreted factors with the potential to modulate lymphocyte viability or function, including enterohemolysin, the StcE metalloprotease, and EspP serine protease (23). Deletion of *toxB* did not appear to affect intestinal colonization of calves by E. coli O157:H7, despite pleotropic effects on the expression and secretion of type III secreted proteins (23, 24). Moreover, although lysates of E. coli O157:H7 were found to inhibit mitogen-activated proliferation of bovine PBMCs, deletion of *toxB* did not fully alleviate this inhibition (15), at least within the limits of the sensitivity of an assay that relied on crude lysates. Here, were definitively show that highly purified recombinant ToxB is a ca. 365-kDa protein capable of concentration-dependent inhibition of ConA-stimulated proliferation of bovine T cells. The ED_50_ for ToxB was calculated to be about 100-fold lower than that seen for lymphostatin tested in parallel, but inhibition was nevertheless detected in the picomolar range, without an apparent cytotoxic effect. The data indicate that LifA and ToxB are part of a family of lymphocyte-inhibitory factors, and further studies are now needed to determine if they act on conserved pathways via shared glycosyltransferase activity. Indeed, a further allelic variant of *toxB* has been described (*toxB2*, distinct from the *toxB1* allele found in serogroup O157 strains [11]), and it will be of interest to determine if such variants share activity and a common mode of action.

Extensive literature has emerged regarding the strategies used by attaching and effacing E. coli to modulate innate immunity, in particular via the activity of type III secreted effectors (25). However, it is becoming clear that AE E. coli also specifically targets the adaptive response. Recently, it was shown that EHEC selectively depletes CD8^+^ T cells in cattle in a manner that requires the locus of enterocyte effacement (26), and the data presented here suggest that lymphostatin and ToxB may act in concert with this strategy to dampen global T cell responses by conditioning T cells to be insensitive to stimuli, likely by modifying cellular factors through glycosyltransferase activity.

## MATERIALS AND METHODS

### Antibodies.

The majority of antibodies used in this study are commercially available or previously described as shown in Table 1.

**TABLE 1:** Antibodies used in this study^*a*^

Specificity	Antibody clone	Purpose	Reference and/or supplier
bo CD3	MM1A	FC	VMRD
bo CD4*	CC30	FC	40
bo CD8*	CC63	FC	40
bo WC-1*	CC15	FC	41
bo γδ-TCR	GB21A	FC	VMRD
Mouse IgG1	IgG1-FITC	FC	Invitrogen
Mouse IgG2a	IgG2a-PE	FC	Invitrogen
Mouse IgG2b	IgG2b-FITC	FC	Invitrogen
bo Ig light chain*	ILA-58	BI/TI	42
Ovine CD335* (cross-reactive)	EC1.1	NKI/TI	30; provided by Timothy Connelly
CD8*	ILA-105	TI	43
CD4*	ILA-12	TI	44
bo IFN-γ	CC330	ELISA	Serotec
bo IFN-γ	CC302b	ELISA	Serotec
bo IL-4	CC313	ELISA	Serotec
bo IL-4	CC314b	ELISA	Serotec
bo IL-10	CC318	ELISA	Serotec
bo IL-10	CC320b	ELISA	Serotec
bo IL-2	AbD14385	ELISA	37; provided by Martin Vordermeier
bo IL-2	Goat serum-b	ELISA	R&D Systems

a Asterisks indicate antibodies produced in-house from hybridomas. bo, bovine; Ig, immunoglobulin; b, biotinylated; FC, flow cytometry; BI, B cell isolation; TI, T cell isolation; NKI, NK cell isolation.

### Expression plasmids and cloning.

The pRham-LifA-6xH plasmid, encoding *lifA* from the prototype EPEC O127:H6 strain E2348/69 under the control of a rhamnose-inducible glucose-repressible promoter, has been described previously (5). The full-length gene encoding ToxB (approximately 9.5 kb) was cloned using the same commercially available Expresso rhamnose cloning and expression system (Lucigen Inc.). The ToxB gene was amplified using genomic DNA from E. coli O157:H7 strain TUV 93-0 (an *stx*_1_ and *stx*_2_ mutant of the prototype EDL933 strain [27]) as a template. Amplicons were generated using the primers ToxB-pRham For (5′ GAA GGA GAT ATA CAT ATG ATT CAT CCT GGC TCT TCT TTA 3′) and ToxB-pRham Rev (5′-GTG ATG GTG GTG ATG ATG CTT TTT TGA GGG GAC AAT 3′). Clones were verified as previously described using Sanger sequencing on both strands and alignment of reads to the EDL933 *toxB* (*l7095*) sequence (5).

### Recombinant protein production and purification.

Recombinant His-tagged lymphostatin (rLifA) was overexpressed in E. cloni cells (Lucigen Inc.) cultured in lysogeny broth at 30°C with shaking at 250 rpm to an absorbance at 600 nm (*A*_600_) of ∼0.8, induced and purified as previously described (5). Recombinant ToxB (rToxB) was overexpressed in E. cloni cells cultured in 2× tryptone yeast (TY) broth. Cells were initially grown at 37°C and 250 rpm to an *A*_600_ of ∼0.4 and cooled to 20°C, and expression was induced by the addition of 0.2% (wt/vol) l-rhamnose once the *A*_600_ reached ∼0.7. Cells were cultured for a further 20 h at 20°C and harvested by centrifugation. Cell pellets were resuspended in 20 mM sodium phosphate (pH 7.5), 300 mM sodium chloride, 500 mM NDSB201, 10% (vol/vol) glycerol, 1 mM dithiothreitol (DTT), 100 μM phenylmethylsulfonyl fluoride, 1 protease inhibitor tablet/3 g of cells (cOmplete, EDTA free; Roche), and 0.2% (vol/vol) Tween 20 and lysed by a single passage at 30 kpsi through a Constant Systems Cell Disruptor TS series benchtop instrument at 6°C (Constant Systems). All purification steps were carried out on ÄKTAexplorer 10 (GE Healthcare) equipment at 6°C. Cell lysates were clarified by centrifugation and purified using a Co^2+^ ion-metal affinity chromatography (HisTrap FF Crude 5 ml; GE) column, preequilibrated in buffer A (20 mM sodium phosphate [pH 7.5], 300 mM sodium chloride, 10% [vol/vol] glycerol, 1 mM DTT, 0.2% [vol/vol] Tween 20). rToxB was eluted by increasing the concentration of buffer B (20 mM sodium phosphate [pH 7.5], 300 mM sodium chloride, 250 mM imidazole, 10% [vol/vol] glycerol, 1 mM DTT, 0.2% [vol/vol] Tween 20) over a 10-column volume (CV) linear gradient from 0 to 100%. Fractions containing rToxB were buffer exchanged into 10 mM Tris (pH 7.5), 50 mM sodium chloride, 10% (vol/vol) glycerol, 1 mM DTT, and 0.2% (vol/vol) Tween 20 (buffer C) and further purified by strong anion-exchange chromatography (IEX) (HiPrep desalt 26/10; RESOURCE Q 6 ml; GE). The protein was eluted by increasing the concentration of buffer D (10 mM Tris [pH 7.5], 1 M sodium chloride, 10% [vol/vol] glycerol, 1 mM DTT, 0.2% [vol/vol] Tween 20) over a 20-CV linear gradient from 0 to 50%. rToxB was eluted at a concentration of ∼190 mM sodium chloride. The sample was concentrated to ∼5 mg/ml of total protein (vivaspin20; 30-kDa molecular mass cutoff; 4,000 × *g*; Sartorius) and passed over a size exclusion column at 1 ml/min preequilibrated in buffer E (20 mM Tris [pH 7.5], 200 mM sodium chloride, 10% [vol/vol] glycerol, 1 mM DTT, 0.2% [vol/vol] Tween 20) to separate low-molecular-weight contaminants (Superose-6pg XK16/60; GE). As a final polishing and concentration step, the fractions containing rToxB were bound to a high-performance strong IEX column (Mono Q 5/50 GL; GE), after exchange into buffer C as previously described, and eluted over a 20-CV linear gradient, 0 to 50% buffer D. The protein was eluted at a salt concentration of 230 mM. The final yield of recombinant ToxB was very low (∼130 μg from 10 liters of culture).

### Isolation of PBMCs from bovine blood.

Access to bovine blood for these studies was approved by the local animal welfare and ethical review body, and blood collection was carried out in accordance with the Animals (Scientific Procedures) Act, 1986. Peripheral blood mononuclear cells were isolated from Holstein-Friesian cattle aged 12 to 18 months old. Briefly, blood was collected into citrate phosphate dextrose in bags or syringes. Following centrifugation at 1,200 × *g* for 15 min to generate an initial buffy coat, the white blood cell fractions were pooled and layered over Ficoll-Paque Plus (GE Healthcare) and centrifuged for 30 min at 1,200 × *g* with the brake off. The PBMCs were collected from the interface and washed several times before use and then further purified depending on the cell population of interest.

### Cell isolation methods.

The T cell fraction was enriched from isolated PBMCs using a sterile wool column (Polysciences, Inc.), as previously described (5). The B cell fraction was magnetically sorted from PBMCs using an anti-bovine Ig light chain antibody (28) (Table 1) and magnetically activated cell sorting (MACS) LS columns (Miltenyi Biotec) by following the manufacturer's instructions. The purity of cells was assessed by single-channel flow cytometry and was >95%. The NKp46^+^ (CD335) NK cell fraction was isolated using magnetic sorting and positive selection from PBMCs as previously published (29), using antibody EC1.1 (30), and transferred into medium lacking IL-2 overnight. Briefly, cells were seeded at 5 × 10^5^ cells/ml in 24-well tissue culture treated plates in RPMI 1640 medium containing 10% (vol/vol) fetal bovine serum (FBS), 100 U/ml of penicillin-streptomycin, 2 mM l-glutamine, 0.1 mM nonessential amino acids, 1 mM sodium pyruvate, and 50 μM beta-mercaptoethanol and left overnight at 37°C in a 5% CO_2_ humidified atmosphere to allow disassociation of the magnetic beads. Beads and bead-associated cells were removed using a magnet. The remaining cells were collected, washed, counted, seeded at 2 × 10^5^ cells/well in 96-well plates, stimulated with bovine IL-12 and -18 (produced in-house [31]) at concentrations of 20 biological units (BU)/ml and 20 ng/ml, respectively, with and without lymphostatin, as indicated, and incubated at 37°C in a 5% CO_2_ atmosphere (32). After 24 h, the cell-free supernatant was collected from the plates and frozen at −20°C until assayed. The purity of cells was evaluated by single-channel flow cytometry and was on average 85%.

### T and B cell stimulation.

Except where indicated, T cells were stimulated with the mitogen concanavalin A (ConA) at a final concentration of 1 μg/ml as previously described (5). rLifA was added at a final concentration and time as indicated in the figure legends in a final volume of 100 μl/well. Where cells were pretreated with recombinant protein, cells were spun down and washed twice with phosphate-buffered saline (PBS) to remove any remaining protein before addition of the stimulant. The carrier buffer for rLifA was determined to have no effect on ConA stimulation of cells (data not shown). Where required, phorbol 12-myristate 13-acetate (PMA) and ionomycin were used to treat T cells at 50 ng/ml and 1 μg/ml, respectively. For IL-2 stimulation, PBMCs were cultured at 10^6^/ml in medium (Iscove's modified Dulbecco's medium, 10% [vol/vol] FBS, 50 μM beta-mercaptoethanol, 50 μg/ml of gentamicin) for 96 h with 5 μg/ml of ConA to upregulate the IL-2 receptor as previously described (33). Cells were taken up and washed in buffer (Hanks balanced salt solution, 2% [vol/vol] FBS, 10 U/ml of sodium heparin, 50 μg/ml of gentamicin, 0.05% [vol/vol] beta-mercaptoethanol). Cells were then plated in 96-well flat-bottom plates as described above, treated with lymphostatin, and stimulated with tissue culture supernatant containing recombinant bovine IL-2 (provided by S. Wattegedera, Moredun Research Institute) for 72 h at 37°C in a 5% CO_2_ humidified atmosphere. Measurements of the ConA-stimulated response were typically 2- to 5-fold higher than for cells alone. B cells were assayed for IL-4-stimulated proliferation similarly to T cells. Briefly, cells were incubated with a 1/50 dilution of bovine IL-4 containing tissue culture supernatant (produced in-house and titrated for optimal activity [34]), with and without recombinant lymphostatin, for a total of 72 h. For all of these assays, at 18 h before the end of incubation, the colorimetric substrate CellTiter 96 AQueous One (Promega) was added to all wells, and optical density measurements were subsequently carried out at 492 nm on a Multiskan Ascent plate reader (Thermo Scientific). Absorbance readings for IL-4 stimulation were typically more than 2-fold higher than for cells alone. Cells and medium alone were used as negative controls. Background medium measurements were subtracted from all values. The response of stimulated lymphocytes to treatments is expressed as a proliferation index, calculated as follows: absorbance (ratio of treatment minus background)/absorbance (mitogen, antigen, or cytokine alone minus background). For all proliferation experiments, cells alone with no treatment, cells treated with the stimulus, and cells treated with protein carrier buffer plus stimulus were included as controls. The ratio of the absorbance readings of stimulus minus treatment/cells alone was used to verify that each individual assay was successful.

### Antigen-specific stimulation of bovine CD4 and CD8 T cells.

A Holstein-Friesian calf was immunized against the protozoan parasite Theileria parva, the causative agent of East Coast fever, by simultaneous subcutaneous administration of cryopreserved infectious sporozoites and long-acting oxytetracycline (20 mg/kg) as described elsewhere (17). PBMCs were isolated 6 weeks postimmunization and were stimulated at 7-day intervals by coculture with a gamma-irradiated (60 Gy) transformed cell line permanently infected with T. parva. This transformed line was established by *in vitro* infection of autologous naive PBMCs with T. parva sporozoites as described elsewhere (35), and such lines express high levels of major histocompatibility complex class I (MHC-I) and MHC-II and effectively act as antigen-presenting cells for T. parva antigens. At day 21, CD4- and CD8-positive T cells were purified by fluorescence-activated cell sorting (FACSAria III; BD Biosciences) by negative selection using monoclonal antibodies specific for γδ-TCR and either anti-CD8 or anti-CD4 (Table 1). Purified T cell lines were maintained in culture (supplemented with recombinant human IL-2 [rhIL-2; Proleukin, Novartis] at 100 U/ml) by weekly restimulation with infected cells as described above, at a 1:1 ratio for CD4 T cells and a 5:1 ratio for CD8 T cells. The purity of T cell lines was assessed by immunofluorescence staining and flow cytometry (FACS Calibur; BD Biosciences) immediately prior to use, and the lines were confirmed to be >99% pure CD4 or CD8 T cells.

### IFN-γ ELISPOT assay.

IFN-γ-producing cells were assessed by ELISPOT assay according to the principles described in reference 36. Briefly, 96-well multiscreen-HA 45-μm plates (Merck Millipore) were coated overnight with 8 μg/ml of anti-bovine IFN-γ capture antibody (Table 1). After washing and blocking for 2 h with RPMI medium supplemented with 10% (vol/vol) FBS, cells and reagents were set up. Heterogeneous CD4^+^ or CD8^+^ T cell populations derived from a single donor as described above were plated on the prepared plates at a density of 10^5^ cells/well at day 7 after treatment with rhIL-2 in RPMI medium supplemented with 10% heat-inactivated FCS, 100 U/ml of penicillin, 100 μg/ml of streptomycin, 2 mM glutamine, and 50 μM 2-mercaptoethanol. Cells were incubated with recombinant lymphostatin and stimulated with irradiated autologous T. parva-infected cells as antigen-presenting cells (APCs) at a ratio of 10:1 T cells to irradiated stimulators. Cells were incubated overnight at 37°C in a 5% CO_2_ humidified atmosphere. Plates were incubated for ∼18 h at 37°C before washing and addition of 5 μg/ml of biotinylated anti-bovine IFN-γ detection antibody (Table 1). Plates were incubated for 90 min with Vectastain ABC (peroxidase standard; Vector Laboratories), followed by development with 3-amino-9-ethylcarbazole (AEC) substrate solution (Merck Millipore). The reaction was stopped with copious quantities of water, and plates were dried and then read on an automated plate reader (Advanced Imaging Devices) using ELISPOT 7.0 Ispot software (Advanced Imaging Devices). Wells with T cells alone, irradiated stimulators alone, and protein alone were included as negative controls.

### Flow cytometry.

Cells were stained with antibodies as indicated in Table 1. Where appropriate, secondary staining with an appropriate antibody-coupled fluorophore was carried out. All samples were analyzed on a FACSCalibur using CellQuest (BD Biosciences) and FlowJo software (Tree Star). A minimum of 20,000, and up to 50,000, events were collected with an initial gate for live cells based on forward/side scatter parameters.

### ELISAs.

Bovine cytokines were measured using a standard sandwich enzyme-linked immunosorbent assay (ELISA) technique. The same method was used for IFN-γ, IL-4, and IL-10. Briefly, 96-well plates (Immunosorb; Nunc) were coated with capture antibody as indicated in Table 1 and incubated at either ambient temperature or 4°C overnight. Plates were washed 5 times with wash buffer (PBS–0.05% [vol/vol] Tween 20) and blocked for 2 h at ambient temperature in PBS containing 1 mg/ml of sodium casein. Plates were washed 5 times in wash buffer. Supernatants were added either neat (T cell cytokine secretion) or at an appropriate dilution to fall on the standard curve (NK cells), incubated for 2 h at ambient temperature, and washed again five times with wash buffer, and detection antibody was added at the concentration indicated in Table 1. Plates were incubated for 1 h and washed again five times in wash buffer, and streptavidin-horseradish peroxidase (HRP) conjugate was added. Plates were incubated for 90 min and washed a final five times in wash buffer. Signal was developed using a 3,3′,5,5′-tetramethylbenzidine (TMB) substrate solution (BioLegend). Optical density measurements were carried out at 450 nm on a Multiskan Ascent plate reader (Thermo Scientific). Bovine IL-2 was measured using a previously published protocol (37), antibody was kindly provided by Martin Vordermeier (Animal & Plant Health Agency, UK), and bovine IL-17A was measured using a commercially available kit (Kingfisher Biotech).

### Statistical analysis.

Calculation of the effective dose required to inhibit cell proliferation by 50% (ED_50_) was carried out using the drm function in the drc package using R (38). All other statistical analysis as indicated was carried out using Minitab (39). *P* values of ≤0.05 were taken to be significant.
